# Cytoprotective role of human dental pulp stem cell-conditioned medium in chemotherapy-induced alopecia

**DOI:** 10.1186/s13287-024-03695-3

**Published:** 2024-03-18

**Authors:** Hui Chen, Satoshi Yamaguchi, Yilin Wang, Kento Kaminogo, Kiyoshi Sakai, Hideharu Hibi

**Affiliations:** 1 Department of Oral and Maxillofacial Surgery, Nagoya University Graduate School of Medicine, Nagoya, Japan; 2 Department of Oral and Maxillofacial Surgery, Nagoya University Hospital, 65 Tsurumai-cho, Showa-ku, Nagoya, Aichi 466-8550 Japan

**Keywords:** Alopecia, Chemotherapy, Mesenchymal stem cell, Conditioned medium, Cytoprotection

## Abstract

**Background:**

Chemotherapy-induced alopecia (CIA) is a distressing adverse effect of chemotherapy, with an estimated incidence of 65% and limited treatment options. Cyclophosphamide (CYP) is a common alopecia-inducing chemotherapy agent. Human dental pulp stem cells (DPSCs) secrete several paracrine factors that up-regulate hair growth. Conditioned medium (CM) collected from DPSCs (DPSC-CM) promotes hair growth; culturing mesenchymal stem cells under hypoxic conditions can enhance this effect.

**Methods:**

The effect of DPSC-CM cultured under normoxic (N-) and hypoxic (H-) conditions against CYP-mediated cytotoxicity in keratinocytes was examined using cell viability assay, lactate dehydrogenase (LDH) cytotoxicity assay, and apoptosis detection. The damage-response pathway was determined in a well-established CIA mouse model by analyzing macroscopic effects, histology, and apoptosis. Reverse transcription-quantitative PCR and Caspase-3/7 activity assay were used to investigate the impact of DPSC-CM on the molecular damage-response pathways in CYP-treated mice. The effect of post-CIA DPSC-CM application on post-CIA hair regrowth was analyzed by macroscopic effects and microstructure observation of the hair surface. Furthermore, to investigate the safety of DPSC-CM as a viable treatment option, the effect of DPSC-CM on carcinoma cell lines was examined by cell viability assay and a subcutaneous tumor model.

**Results:**

In the cell viability assay, DPSC-CM was observed to increase the number of keratinocytes over varying CYP concentrations. Furthermore, it reduced the LDH activity level and suppressed apoptosis in CYP-treated keratinocytes. DPSC-CM exhibited the cytoprotective role in vivo via the dystrophic anagen damage-response pathway. While both N-CM and H-CM downregulated the Caspase-3/7 activity level, H-CM downregulated Caspase-3 mRNA expression. The proportion of post-CIA H-CM-treated mice with > 90% normal hair was nearly twice that of vehicle- or N-CM-treated mice between days 50 and 59 post-depilation, suggesting that post-CIA H-CM application may accelerate hair regrowth and improve hair quality. Furthermore, DPSC-CM suppressed proliferation in vitro in certain carcinoma cell lines and did not promote the squamous cell carcinoma (SCC-VII) tumor growth rate in mice.

**Conclusions:**

The potentiality of DPSC-CM and H-CM as a promising cytoprotective agent and hair regrowth stimulant, respectively, for CIA needs in-depth exploration.

**Supplementary Information:**

The online version contains supplementary material available at 10.1186/s13287-024-03695-3.

## Background

Chemotherapy-induced alopecia (CIA) is a disturbing adverse effect of chemotherapy; because of the extreme anxiety of hair loss, 8% of female patients may avoid chemotherapy [1]. Notably, CIA has an estimated incidence of 65%, depending on the chemotherapeutic protocol [2]. Permanent CIA (pCIA) characterized by the absence of hair regrowth > 6 months after treatment discontinuation is increasingly being reported [3–6]. Cyclophosphamide (CYP), busulfan, anthracycline, carboplatin, docetaxel, paclitaxel, and etoposide are the most common pCIA-inducing chemotherapeutics [5, 6]. Moreover, hair thinning and hair discoloration are common and persistent long-term problems [4]. Lasting hair thinning and discoloration, as well as the occurrence of pCIA, highlight the importance of further research into effective CIA treatments. To date, only scalp cooling has been approved by the United States Food and Drug Administration in 2015 as a preventive treatment for CIA [7]. This treatment has proven reasonably effective, despite side effects such as headache, dizziness, and scalp pain [8], while many other treatment options are unsatisfactory [9]. Thus, exploring a novel treatment for CIA is a crucial concern.

Human dental pulp stem cells (DPSCs) are mesenchymal stem cells (MSCs) with self-renewal capabilities and multi-lineage differentiation residing in the perivascular niche of the dental pulp [10]. The reparative role of these cells is mainly attributed to paracrine factors in the cell culture medium or conditioned medium (CM) [11]. Cell-free CM-based therapy has several advantages over MSC-based therapies, including higher safety in terms of tumorigenicity, immune compatibility, transmission of infections, and emboli formation; possible use in allogeneic therapies; and easier production, evaluation, storage, and transportation, enabling the production of CM into ready-to-go biologic products for regenerative medicine [12–14]. DPSC-CM has several promising applications in cardiac injury [15], bone healing [16], liver injury [17], and diabetes [18]. CM collected from DPSCs or other sources of MSCs promotes hair growth [14, 19–22] because it contains paracrine factors that up-regulate hair growth, such as keratinocyte growth factor (KGF), vascular endothelial growth factor (VEGF), hepatocyte growth factor (HGF), platelet-derived growth factor (PDGF), insulin-like growth factor I (IGF-I), and basic fibroblast growth factor (bFGF) [23, 24], and this effect can be enhanced by an increase in the relevant paracrine factors, such as VEGF, bFGF, and PDGF, by culturing MSCs under hypoxic conditions in dermal papilla and outer root sheath cells, organ culture, and in mice [19, 20]. A previous study revealed that hypoxia enhanced the hair growth-promoting effects of embryonic stem cell-derived MSC-CM via NADPH oxidase 4 [19]. In another study, hypoxia treatment of adipose-MSCs promoted the growth of dermal papilla cells via hypoxia-inducible factor-1α and ERK1/2 signaling pathways [20]. Moreover, previous studies have highlighted the superiority of CM collected from DPSCs over that from other sources of MSC for similar applications [11, 15, 25, 26].

Newer treatment options for CIA need to overcome two apparently paradoxical response pathways in chemotherapy-damaged hair follicles (HFs). Mild chemotherapy-induced toxicity initiates the dystrophic anagen response pathway with decelerated hair regrowth, whereas more severe toxicity initiates the dystrophic catagen response pathway with accelerated hair regrowth [9, 27, 28]. In the dystrophic anagen response pathway that is characterized by less alopecia, the damaged HF remains longer in the same anagen phase than normal HFs, producing poor-quality and depigmented hair shafts during primary recovery, before proceeding to reconstruct normal hair shafts during the secondary recovery; if the dystrophic catagen response pathway, characterized by more alopecia, is initiated, the damaged HF immediately transitions from the anagen phase into the dystrophic catagen phase, followed by a shortened telogen phase, and subsequently, begins to produce secondary recovery hair shafts [29]. The principle that milder toxicity leads to lower damage, less hair loss, and retarded hair regrowth is crucial for understanding CIA pathophysiology.

In this study, we aimed to investigate the role of CM collected from DPSCs cultured under normoxic (N-) and hypoxic (H-) conditions in CIA treatment. To provide more possibilities for applications of DPSC-CM and offer novel therapeutic strategies for CIA, we tested the effects of DPSC-CM against CYP-mediated cytotoxicity in normal human epidermal keratinocytes (NHEKs) and in a well-established CIA mouse model [27, 29, 30]. Furthermore, to investigate the safety of DPSC-CM as a viable treatment option, we examined its effect on carcinoma cell lines in vitro and in vivo.

## Methods

### DPSCs culture and CM preparation

DPSCs (Lonza, Walkersville, USA) were characterized by surface marker profiling (negative: CD34, CD45, and CD133; positive: CD105, CD166, CD29, CD90, and CD73) performed by the manufacturer. DPSCs at passage 6–8 were cultured in Dulbecco’s Modified Eagle’s Medium (DMEM; Sigma-Aldrich, Saint Louis, USA) supplemented with 10% fetal bovine serum (Bovogen, Melbourne, Australia) and 1% penicillin–streptomycin solution (Fujifilm Wako, Osaka, Japan) at 37 °C in 5% CO_2_ until 80% confluency. Subsequently, these cells were washed with phosphate-buffered saline (PBS; Fujifilm Wako, Osaka, Japan), and the culture medium was replaced with the vehicle medium (serum-free DMEM). After 48 h of incubation in 21% and 1% O_2_ (normoxic [N-] and hypoxic [H-] conditions, respectively), the cells were collected and centrifuged at 400 × g and 4 °C for 3 min. The supernatants were collected and centrifuged at 1700 × g and 4 °C for 3 min. Following this, the supernatants resulting from this centrifugation were collected and defined as N-CM or H-CM. CM was stored at − 80 °C before being used in the subsequent experiments.

### In vitro model: role of DPSC-CM in CYP-induced keratinocyte cytotoxicity

The NHEKs (Kurabo, Osaka, Japan) were cultured in a keratinocyte serum-free medium (KSFM; HuMedia-KG2, Kurabo, Osaka, Japan) according to the manufacturer’s instructions. They were used at passage 1–3 to ensure maximal proliferative capacity for all assays.

The NHEKs were seeded in 96-well culture plates and pre-treated with a mixture of 75 uL KSFM and 75 uL control-group medium (DMEM) or test-group media, i.e., vehicle (DMEM), N-CM, or H-CM, for 24 h in each well. These cells, except those in the control group, were subsequently stimulated by CYP (Endoxan, Shionogi, Osaka, Japan) for 48 h before being assayed; 50 µL of CYP dissolved in KSFM was added directly to the previous culture medium in each well. To avoid cell loss, NHEKs were centrifuged at 1500 RPM for 3 min, in the culture plates, before medium change or addition.

### Cell viability assay

Cell viability was tested using the Cell Counting Kit-8 (Dojindo, Kumamoto, Japan). The 96-well culture plates were incubated for 2–3 h after adding Cell Counting Kit-8. The absorbance at 450 nm was subsequently measured using a microplate reader (Infinite M200pro, Tecan, Männedorf, Switzerland). Representative photomicrographs were taken (EVOS FL, Thermo-Fisher, Waltham, USA).

### Lactate dehydrogenase cytotoxicity assay

The activity level of lactate dehydrogenase (LDH) released through the damaged plasma membrane of the NHEKs into the supernatant was assayed using the Cytotoxicity LDH Assay Kit-WST (Dojindo, Kumamoto, Japan). The culture supernatant was used to measure LDH, and the cells were used in the cell viability assay. The absorbance of the supernatant or the cells was subsequently measured using a microplate reader (Infinite M200pro, Tecan, Männedorf, Switzerland). The LDH activity level was defined as the ratio of the supernatant absorbance to the living cell absorbance. Each independent experiment comprised six technical replicates.

### Determination of apoptosis

An In Situ Cell Death Detection Kit, TMR red (Roche, Basel, Switzerland) was used for terminal deoxynucleotidyl transferase dUTP nick end labeling (TUNEL) staining, while DAPI (R37606, Life Technologies, Carlsbad, USA) was used for nuclear staining. The images were taken using a fluorescence microscope (BZ-X800, Keyence, Osaka, Japan).

In vitro: Cells were counted automatically using a BZ-X800 Analyzer (Keyence, Osaka, Japan). In each independent experiment, 20 different fields in one well of a 96-well plate were analyzed for one group.

In vivo: TUNEL staining was performed on 4-μm thick formalin-fixed paraffin-embedded sections. The skin tissue was divided equally into three sections (head, middle, and tail) for analysis. A minimum of 95 HFs from the dorsal skin at 100–200 × magnification was analyzed for each mouse.

### In vivo CIA model

Six-week-old syngeneic female C57BL/6J mice were purchased from Charles River Japan (Tokyo, Japan). The mice were housed in standard cages on a 12 h light/dark cycle and were provided water and mouse chow ad libitum.

To minimize fluctuations in the HF cycle, dorsal skin HFs in the telogen phase were induced to enter the anagen phase immediately via depilation with a wax sheet (Veet, Reckitt Benckiser, Tokyo, Japan), confirmed by a pink skin color after a 7-day acclimatization period [31]. A single dose of 120 mg/kg CYP dissolved in 0.9% NaCl solution was injected intraperitoneally on day 9 post-depilation (p.d.) [29]. The mice were administered inhalation anesthesia (apparatus: Narcobit-e II, Natsume Seisakusho, Tokyo, Japan) before all procedures.

In the mouse model aimed at studying CIA progression and the damage-response pathway, a total of 54 mice, 18 in each group, were used. The vehicle (DMEM), N-CM, or H-CM were injected subcutaneously once a day from day 2 p.d. to days 14, 16, or 32 p.d., respectively, when the mice were euthanized for various purposes. The mice were euthanized with a carbon dioxide inhalation device and the fill rate of carbon dioxide was standardized by the experimental animal division of Nagoya University. If both cessation of breathing and ocular pallor were observed after initiation of carbon dioxide inhalation, the mice would continue to be exposed to carbon dioxide for an additional passive exposure time of 3 min, which reliably resulted in irreversible euthanasia for mice.

In the mouse model aimed at studying post-CIA hair regrowth, a total of 18 mice, 6 in each group, were used. The vehicle (DMEM), N-CM, or H-CM were injected subcutaneously daily from day 15 p.d. to day 59 p.d. when the mice were euthanized by carbon dioxide inhalation. The microstructure of the hair surface was observed using a scanning electron microscope (SEM).

### Macroscopic analysis

Images of mouse dorsal skin were first converted to 8-bit format and subsequently analyzed for CIA and hair regrowth using ImageJ (National Institutes of Health, Bethesda, USA). The grayscale (0–255) threshold was uniformly set, with 0–35 and 36–255 deemed as dorsal skin with black hair and with alopecia or gray hair, respectively. Before day 16 p.d., the mice had almost no gray hair, and all hair shafts were black. Here, areas of grayscale 0–35 and 36–255 were considered as dorsal skin with hair and alopecic skin, respectively, before day 16 p.d..

### Quantitative histological analysis

Histological analysis of HFs was performed in sections stained with hematoxylin and eosin. Fresh skin tissue was washed with PBS and fixed with 10% formalin neutral buffer solution for 24 h. Tissue specimens were automatically processed for 21 h by a Tissue-Tek VIP 6 tissue processor (Sakura, Tokyo, Japan) and subsequently embedded in paraffin. The samples were cut equally into three sections (head, middle, and tail) and sliced into 4-μm slices before deparaffinization, hydration, and staining. A minimum of 78 dorsal skin HFs at 100–400 × magnification was analyzed for each mouse. The HF was identified and classified according to its hair cycle stage and signs of follicle dystrophy [28].

### Determination of mRNA expression

Fresh skin samples were washed with PBS, transferred directly into a pre-chilled (2–8 °C) RNAlater Stabilization Solution (Invitrogen, Waltham, USA), and stored at 4 °C for 48 h. The samples were homogenized automatically using a Multi-Beads Shocker (Yasui, Osaka, Japan) in the presence of liquid nitrogen. For reverse transcription-quantitative PCR (RT-qPCR), the total RNA was extracted using TRIzol reagent (Life Technologies, Carlsbad, USA). Reverse transcription was performed using ReverTra Ace qPCR RT Master Mix with gDNA Remover (Toyobo, Osaka, Japan), while RT-qPCR was performed using THUNDERBIRD SYBR qPCR Mix (Toyobo, Osaka, Japan) driven by the AriaMx Real-Time PCR System (Agilent, Santa Clara, USA). *Gapdh* was used as the reference gene. The primers, designed by Primer Premier 5 (Premier Biosoft, San Francisco, USA), are listed in Additional file 1: Table S1.

### Caspase-3/7 activity assay

Fresh skin samples were washed with PBS, transferred directly into a pre-chilled (2–8 °C) MACS Tissue Storage Solution (Miltenyi Biotec, Bergisch Gladbach, Germany), and stored at 4 °C for 24 h. The samples were homogenized automatically using the Multi-Beads Shocker (Yasui, Osaka, Japan) in the presence of liquid nitrogen and subsequently using an ultrasonic homogenizer (As One, Osaka, Japan), with a 20 × cell lysis buffer (200 mM TRIS, pH 7.5, 2 M NaCl, 20 mM EDTA, and 0.2% TRITON X-100). Thereafter, the samples were diluted 20 × to 1 ×. Next, the protein concentration of the samples was adjusted to a uniform concentration using the BCA Protein Assay Kit (Pierce, Thermo Scientific, Waltham, USA). Caspase-3/7 activity was assayed using the EnzChek Caspase-3 Assay Kit #1 (Molecular Probes, Eugene, USA). The fluorescence (excitation/emission 342/441 nm) was measured by a fluorescence microplate reader (Cytation 5, Biotek, Winooski, USA).

### Microstructure observation of the hair surface

The dried hair specimens were fixed onto a brass stub before being coated with an osmium plasma coater (NL-OPC80NS, Nippon Laser &Electronics, Nagoya, Japan). Microscopic observation was performed at 1000 × magnification with a SEM (JSM-7610F, JEOL, Tokyo, Japan). The dorsum of each mouse was divided equally into six regions, and a representative hair shaft from each region was selected for analysis.

### Subcutaneous tumor model

Seven-week-old syngeneic female C3H/HeNCrl mice were procured from Charles River Japan (Tokyo, Japan). After a 1-week acclimatization period, 1.08 × 10^6^ poorly immunogenic murine oral squamous cell carcinoma (SCC-VII) cells in 100 uL PBS were subcutaneously inoculated into the dorsal region of the mice. The initiation of the experiment was marked as day 0, corresponding to the point at which the tumor volume (1/2 × long diameter × short diameter^2^) reached 150 mm^3^. On days 0, 3, and 6, the mice were administered 300 uL of the vehicle (DMEM), N-CM, or H-CM via tail vein injections. Tumor volume was measured every 3 days. The mice were euthanized by carbon dioxide inhalation on day 21, and the tumors were resected and weighed.

### Statistical analysis

Data were presented as mean ± SEM of at least three independent experiments. Statistical analysis was performed using Graphpad Prism, version 8.4.0 (Graphpad Software, Boston, USA). Group differences were analyzed by the Mann–Whitney U-test. A value of *P* < 0.05 was considered statistically significant.

## Results

### Cytoprotective role of DPSC-CM against CYP-mediated cytotoxicity in NHEKs

The cytotoxicity of CYP and the effect of DPSC-CM on CYP-mediated cytotoxicity in NHEKs were examined via cell viability and LDH activity assays, as well as TUNEL apoptosis staining. The accuracy and reproducibility of these assays were considerably improved by centrifuging the cells with the culture plates before all medium changes or additions (not depicted herein).

**Fig. 1:**
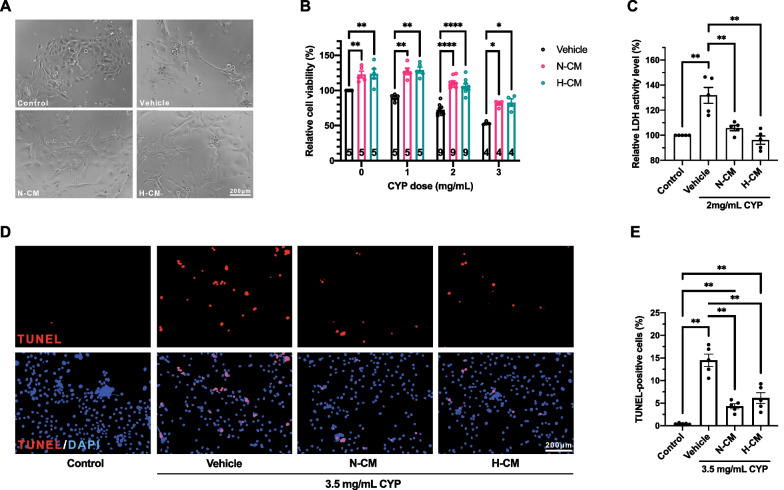
Effect of DPSC-CM on CYP-mediated cytotoxicity in NHEKs. **A** Representative photomicrographs of NHEKs treated with 2 mg/mL CYP in all, except the control group (n = 3 in each group). **B** Cell viability of NHEKs treated with varying CYP concentrations. The numbers at the bottom of the columns represent the number of independent experiments, each comprising six technical replicates. **C** Activity level of LDH released via the damaged plasma membrane of NHEKs into the supernatant (n = 5 in each group). **D** Representative photomicrographs of the TUNEL-stained NHEKs in the quantitative apoptosis analysis of the in vitro CIA model (n = 5 in each group). **E** Quantitative apoptosis analysis of TUNEL-positive NHEKs treated with 3.5 mg/mL CYP in all, except the control group (n = 5 in each group). **A–E** Data are presented as mean ± SEM. *P < 0.05, **P < 0.01, and ****P < 0.0001

In the cell viability assay, representative photomicrographs revealed that compared with the NHEKs in the control group, the cell morphology of the NHEKs in the other groups was altered by CYP treatment (2 mg/mL), transforming from nearly round to elongated, and the NHEKs were scattered more loosely (Fig. 1A). Both N-CM and H-CM increased the number of NHEKs that had been treated with varying CYP concentrations (0, 1, 2, and 3 mg/mL) for 48 h (Fig. 1B). No significant difference was observed between the two groups at all CYP concentrations (Fig. 1B).

The activity level of LDH released via the damaged plasma membrane into the cell culture medium was measured and calculated as described in Methods. N-CM and H-CM reduced the level of LDH activity at a concentration of 2 mg/mL of CYP compared with the vehicle group (Fig. 1C). No significant difference was observed between the two groups (Fig. 1C).

To investigate the effect of DPSC-CM on CYP-mediated apoptosis in the NHEKs, a TUNEL assay was performed. Representative photomicrographs of the TUNEL staining are presented in Fig. 1D. The TUNEL-positive proportions of the N-CM and H-CM groups were lower than that of the vehicle group at a concentration of 3.5 mg/mL of CYP (Fig. 1E). No significant difference was observed in suppression capability between the two groups (Fig. 1E).

Therefore, the outcome of the in vitro experiments strongly demonstrated the cytoprotective role of DPSC-CM against CYP-mediated cytotoxicity in NHEKs.

### DPSC-CM retards the progression of CIA

**Fig. 2:**
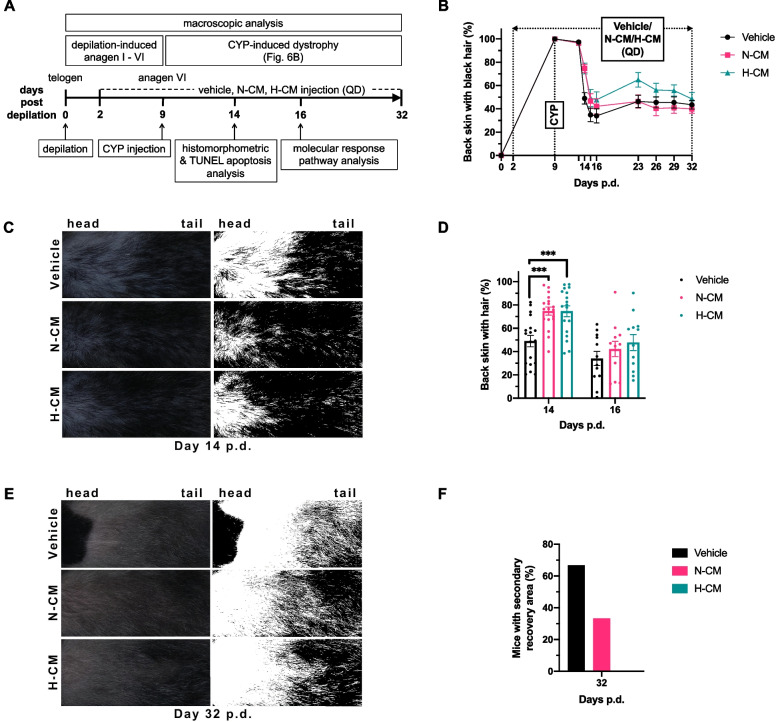
Macroscopic effect of DPSC-CM against CYP-mediated cytotoxicity in mice. **A** Experimental design of the mouse model to study CIA progression and the damage-response pathway. **B** Time course of alopecia and hair regrowth in the mouse model for studying the damage-response pathway. **C** Representative macroscopic effect images of CIA on day 14 p.d. **D** Macroscopic analysis of CIA on days 14 and 16 p.d. **E** Representative macroscopic effect images of post-CIA hair regrowth on day 32 p.d. **F** Macroscopic analysis of regrowth of secondary recovery (black-pigmented) hair shafts in the nuchal dorsal skin on day 32 p.d. (n = 6 in each group). **A–F** Days 0–14 p.d., n = 18 in each group; days 15–16 p.d., n = 12 in each group; and days 17–32 p.d., n = 6 in each group. Data are presented as mean ± SEM. ***P < 0.001. QD, quaque die

The experimental design of the mouse model aimed at studying CIA progression and the damage-response pathway is shown in Fig. 2A. The macroscopic effect of DPSC-CM on the development of CIA and post-alopecia hair regrowth was analyzed as described in Methods. The time course of macroscopic development in the three groups is illustrated in Fig. 2B. Figure 2C presents representative images of the dorsal skin of the mice in the three groups on day 14 p.d., revealing that alopecia first appeared in the area near the head and subsequently progressed to the area near the tail. This nuchal–caudal development of alopecia may be due to the wavelike pattern of spontaneous anagen development post-depilation [31] and the vulnerability to chemotherapy-induced damage that varies with the proliferation rates of HFs [32]. The alopecic area in the dorsal skin of N-CM- or H-CM-treated mice was significantly less than that in the vehicle-treated mice on day 14 p.d., i.e., 5 days post-CYP injection (Fig. 2D). No macroscopic differences were observed between the three groups on day 16 p.d. (Fig. 2D). Thus, while DPSC-CM did not reduce hair loss in CIA, it retarded the progression of CIA.

### Cytoprotective role of DPSC-CM by promoting the dystrophic anagen damage-response pathway

Macroscopic analysis was used initially to determine the damage-response pathway favored by the DPSC-CM. A less alopecic area on day 14 p.d. (Fig. 2B–D) and a lower proportion of mice exhibiting a secondary recovery area (black-pigmented regrown hairs) in the nuchal dorsal skin on day 32 p.d. (Fig. 2E, F) were observed in the N-CM- or H-CM-treated groups than in the vehicle-treated group. These results revealed that DPSC-CM favored the dystrophic anagen damage-response pathway. Representative macroscopic effect images of DPSC-CM on CYP-treated mice on days 14 and 32 p.d. are presented in Fig. 2C and E, respectively.

**Fig. 3:**
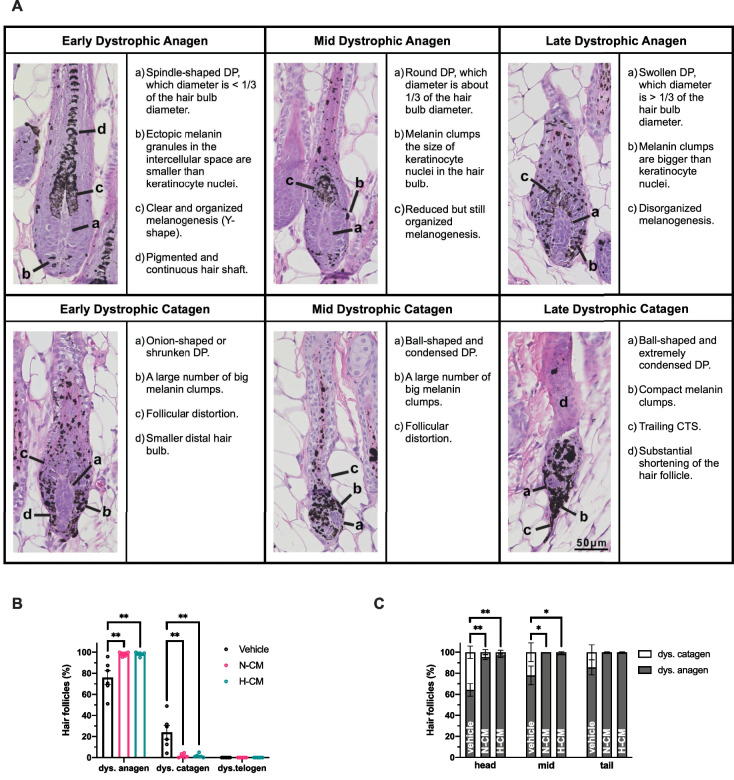
Histomorphometric effect of DPSC-CM against CYP-mediated cytotoxicity in mice. **A** Representative histopathology of dystrophic anagen and catagen HFs on day 14 p.d. HFs were identified and classified according to their hair cycle stage and signs of follicular dystrophy [28]. **B, C** Quantitative histological analysis of HFs for determining the damage-response pathways on day 14 p.d. **A–C** n = 6 in each group. DP, dermal papilla; CTS, connective tissue sheath

To directly determine the damage-response pathway favored by the DPSC-CM, HFs were analyzed via microscopic quantitative histomorphometry on day 14 p.d. The representative histopathology on day 14 p.d. and the classification criteria for HF dystrophy are illustrated in Fig. 3A. The N-CM- or H-CM-treated mice demonstrated a higher proportion of dystrophic anagen HFs than the vehicle-treated mice (Fig. 3B). No significant difference was observed between the two groups (Fig. 3B). In all three groups, no dystrophic telogen HFs were observed (Fig. 3B). Vehicle-treated mice had more HFs in the dystrophic catagen pathway than the N-CM- or H-CM-treated mice (Fig. 3B), especially in the nuchal and middle dorsal skin (Fig. 3C), which was consistent with the distribution pattern of the alopecic areas in Fig. 2C.

**Fig. 4:**
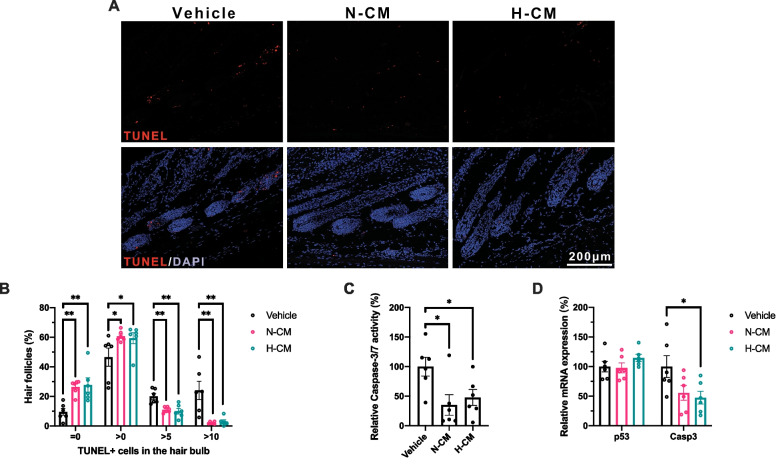
Effect of DPSC-CM on apoptosis due to CYP-mediated cytotoxicity in mouse HFs. **A** Representative photomicrographs of quantitative analysis of TUNEL-positive keratinocytes in the hair bulb on day 14 p.d. **B** Quantitative analysis of TUNEL-positive keratinocytes in the hair bulb on day 14 p.d. **C** Analysis of Caspase-3/7 activity in mice skin tissue on day 16 p.d. **D** CYP-mediated mRNA expression of *p53* and *Casp3* in mice skin tissue on day 16 p.d. **A–D** n = 6 in each group. Data are presented as mean ± SEM. *P < 0.05 and **P < 0.01

To further determine the damage-response pathway favored by the DPSC-CM, TUNEL, RT-qPCR, and Caspase-3/7 activity assays were performed. Representative microphotographs of the quantitative analysis of TUNEL-positive keratinocytes in the hair bulb on day 14 p.d. are exhibited in Fig. 4A. The N-CM- or H-CM-treated mice had more HFs with 0 or 1–5 TUNEL-positive cells in the hair bulb and fewer HFs with 6–10 or > 10 TUNEL-positive cells in the hair bulb than the vehicle-treated mice on day 14 p.d. (Fig. 4B). Furthermore, N-CM and H-CM downregulated the Caspase-3/7 activity level (Fig. 4C), and H-CM downregulated Caspase-3 mRNA expression as well (Fig. 4D). The aforementioned data indicated that N-CM and H-CM suppressed apoptosis in HFs or skin tissues, further corroborating that both promoted the dystrophic anagen damage-response pathway.

Consequently, the cytoprotective role of DPSC-CM through the dystrophic anagen damage-response pathway was strongly demonstrated and validated from multiple perspectives, including macroscopic analysis, microscopic quantitative histomorphometry, TUNEL apoptosis assay, RT-qPCR, and Caspase-3/7 activity assay.

### Effect of DPSC-CM on the molecular damage-response pathways in CYP-treated mice

P53-dependent apoptosis of hair-matrix keratinocytes is essential to the molecular damage-response pathways in CYP-treated mice [33]. As targets for p53, the caspase family of proteases are crucial mediators of complex apoptosis-associated biochemical events [34]. Caspase-3, which has a substrate specificity for the amino acid sequence Asp-Glu-Val-Asp (DEVD), is a critical factor in apoptosis execution, while Caspase-7 is highly homologous to Caspase-3 [34]. In the present study, we tested Caspase-3 and other DEVD-specific protease (e.g., Caspase-7) activities in mice skin tissue on day 16 p.d. and found that N-CM and H-CM downregulated the Caspase-3/7 activity level (Fig. 4C). No significant difference was observed between the two groups (Fig. 4C). Furthermore, downregulated Caspase-3 mRNA expression was observed in the H-CM group compared with the vehicle group (Fig. 4D). Although no significant difference was observed between the N-CM and H-CM groups, N-CM did not significantly downregulate the Caspase-3 mRNA expression (Fig. 4D). No significant difference was observed in the mRNA expression of p53 between the three groups (Fig. 4D). Hence, DPSC-CM could downregulate apoptosis-related activities in HFs via Caspase-3/7 in CYP-treated mice.

### Application of H-CM after CIA may accelerate hair regrowth and improve hair quality

**Fig. 5:**
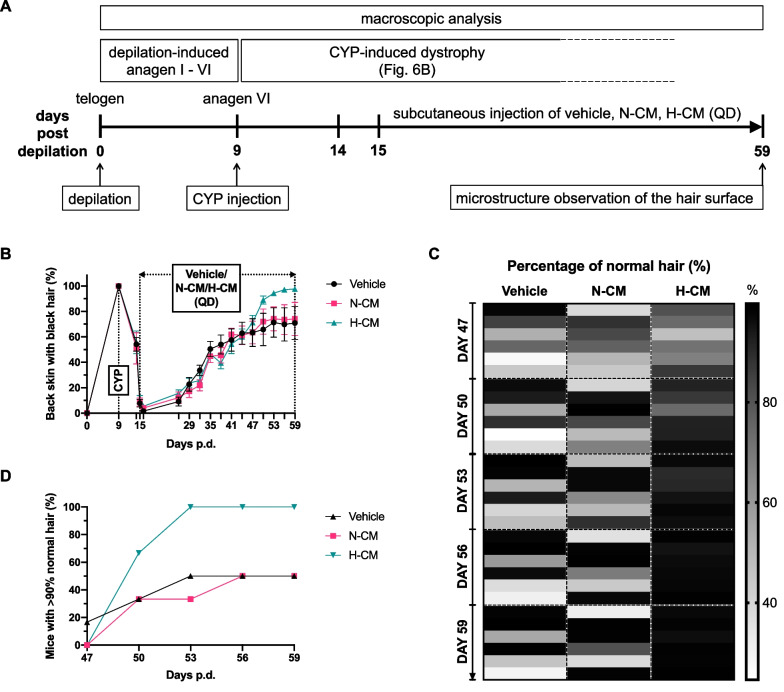
Effect of DPSC-CM applied after CIA on hair regrowth in mice. **A** Experimental design of the mouse model to study post-CIA hair regrowth. **B** Time course of alopecia and hair regrowth in the mouse model for studying post-CIA hair regrowth. Data are presented as mean ± SEM. **C** Heat map of macroscopic analysis of the dorsal area of all mice on days 47–59 p.d. Each small box represents the percentage of normal hair (%) of one mouse. **D** Proportion of mice with > 90% normal hair (secondary recovery hair, i.e., black-pigmented hair) on days 47–59 p.d. **A–D** n = 6 in each group. QD, quaque die

To examine the effect of post-CIA DPSC-CM application on hair regrowth in CYP-treated mice, we adjusted the previous model by delaying treatment initiation with the vehicle, N-CM, or H-CM until day 15 p.d, as illustrated in Fig. 5A. By this time, HFs with more severe toxic effects were already in the dystrophic catagen pathway. This helped avoid interference from differences in the damage-response pathways. The time course of macroscopic development in the three groups is presented in Fig. 5B. No significant difference was observed in the dorsal skin with black hair (%) between the three groups on days 47–59 p.d. (Fig. 5B). A heat map of macroscopic analysis of the dorsal skin of all mice in the three groups on days 47–59 p.d. is exhibited in Fig. 5C. The proportion of H-CM-treated mice with > 90% normal hair (secondary recovery hair, i.e., black-pigmented hair) was nearly twice that of vehicle- or N-CM-treated mice between 50 and 59 days p.d. (Fig. 5D).

**Fig. 6:**
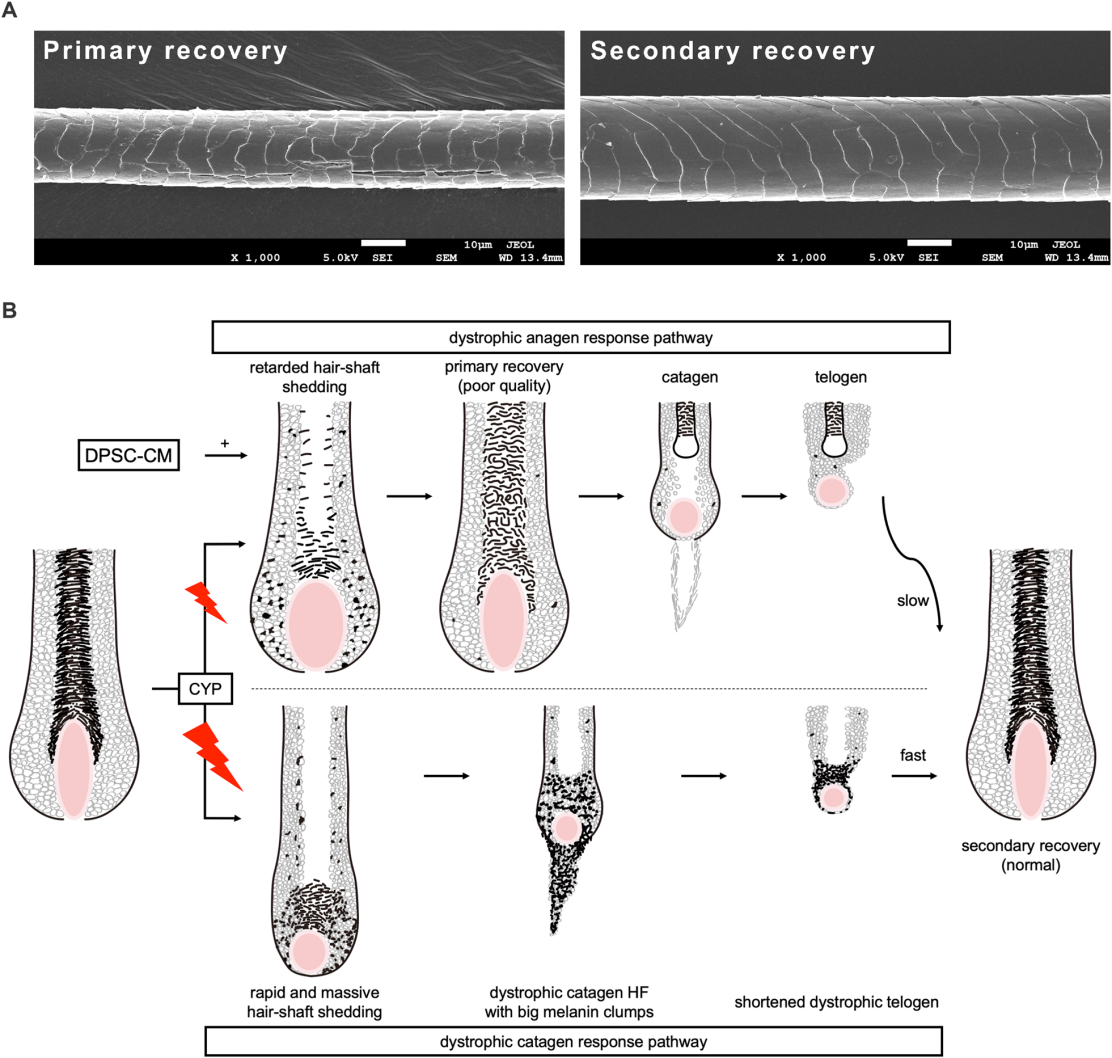
Damage-response pathways of CIA in the hair follicle. **A** Representative scanning electron microscope images of the primary and secondary recovery hair from 18 mice in the vehicle-, N-CM-, and H-CM-treated groups (6 hair shafts/mouse) on day 59 p.d. **B** Two major post-chemotherapy damage-response pathways in the HF: dystrophic anagen and catagen pathways [9, 27–29]. The size of the red flashes indicates the degree of CYP-induced damage to the HF

Microstructure observation of the hair surface on day 59 p.d. was performed using a SEM. Differences were observed between black- and gray-pigmented (secondary and primary recovery hairs, respectively) hair shafts (Fig. 6A). Primary recovery hairs were smaller in diameter and had more surface cracks than secondary recovery hairs, which clearly shows that the quality of the secondary recovery hair was better compared to that of the primary recovery hair (Fig. 6A, B, Additional file 2: Fig. S1).

Therefore, the post-CIA H-CM treatment may accelerate hair regrowth with better hair quality compared with the post-CIA vehicle and N-CM treatment.

### Effect of DPSC-CM on carcinoma cell lines

**Fig. 7:**
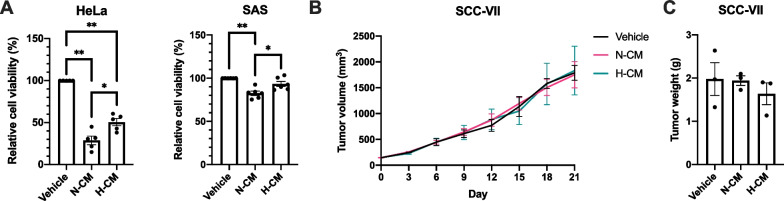
Effect of DPSC-CM on carcinoma cell lines. **A** Cell viability of carcinoma cells in the vehicle (DMEM), N-CM, or H-CM groups after 48 h of incubation. HeLa, n = 5 in each group; SAS, n = 6 in each group. **B**, **C** Tumor volume and tumor weight (day 21) in a subcutaneous tumor model (C3H/HeNCrl mice) that mimics head and neck squamous cell carcinoma (SCC). n = 3 in each group. **A–C** Data are presented as mean ± SEM. *P < 0.05, **P < 0.01

The impact of DPSC-CM on human cell lines derived from cervical cancer (HeLa) and poorly differentiated squamous cell carcinoma of the tongue (SAS) was investigated in vitro in this study. N-CM and H-CM reduced HeLa cell viability after 48 h of incubation compared to the vehicle group (DMEM) (Fig. 7A). N-CM also reduced SAS cell viability after 48 h of incubation compared to the vehicle group (Fig. 7A). Furthermore, N-CM showed a more potent anti-cancer effect than H-CM in HeLa and SAS cells (Fig. 7A). The accuracy and reproducibility of these assays were considerably improved by centrifuging the cells with the culture plates before all medium changes or additions (not depicted herein).

The effect of DPSC-CM on tumor growth rate was examined in a subcutaneous tumor model. SCC-VII in C3H/HeNCrl mice is a tumor model that mimics head and neck squamous cell carcinoma. Tumor volume from day 0 to day 21 and tumor weight on day 21 were measured to reflect the tumor growth rate. There was no significant difference in tumor volume and weight between the vehicle, N-CM, and H-CM groups (Fig. 7B, C).

Thus, DPSC-CM suppressed proliferation in certain carcinoma cell lines in vitro and did not promote SCC-VII tumor growth rate in mice.

## Discussion

This study provides compelling evidence that DPSC-CM application exhibited a cytoprotective role against CYP-mediated cytotoxicity in vitro. Furthermore, it reduced CYP toxicity to HFs and retarded alopecia by promoting the dystrophic anagen damage-response pathway in a CIA mouse model. Moreover, post-CIA H-CM application may accelerate hair regrowth and improve hair quality. However, reducing the toxicity of chemotherapeutic agents on HFs while accelerating post-CIA hair regrowth according to the two damage-response pathways of CIA may prove challenging (Fig. 6B). Therefore, a single goal, such as reducing toxicity or promoting hair regrowth, may be more feasible. This study posits two novel treatment options for CIA: subcutaneous application of DPSC-CM as a cytoprotective agent to reduce the cytotoxicity of chemotherapeutic agents to HFs initiated before alopecia if the risk of pCIA is high, as is common with high-dose chemotherapy [6], and subcutaneous application of H-CM as a stimulant for post-CIA hair regrowth initiated after alopecia for patients who are anxious about their post-chemotherapy appearance and may become depressed. The appropriate time point for treatment initiation is important for its effectiveness.

The underlying principles of CIA pathobiology are crucial for understanding the effect of CYP on CIA, as well as the role of DPSC-CM. Normal HFs undergo four stages of development and cycling: anagen (growth phase), catagen (regression phase), telogen (resting phase), and exogen (active shedding phase) [31]. Given that HFs exhibit the highest proliferation rates during the anagen phase, anagen HFs become the most sensitive and vulnerable to chemotherapy-induced HF damage, making them the main targets in CIA [9, 27]. Furthermore, the melanocytes within anagen HFs generate substantial amounts of melanin requiring precise transfer to the corresponding HF keratinocyte populations. This precision is another factor that renders anagen HFs and their pigmentary system most sensitive to chemotherapy-induced damage [9, 28, 31]. Thus, anagen effluvium is the most characteristic HF response to CYP-induced damage. In the present study, all HFs of the 6-week-old mice were in the telogen phase after 1-week acclimatization (Figs. 2A, 5A). Following depilation, telogen HFs swiftly transition to the anagen phase, entering anagen VI on day 9 p.d.—a suitable time for CYP injection [31]. After the CYP administration, HFs enter the dystrophic anagen response pathway or the dystrophic catagen response pathway depending on the degree of toxicity (Fig. 6B). In the mouse model aimed at studying CIA progression and the damage-response pathway, we analyzed the ratio of the two response pathways in the HFs using hematoxylin and eosin staining; the results of the macroscopic analysis and the apoptosis-related analysis further validated the cytoprotective role of DPSC-CM in CIA through the dystrophic anagen response pathway (Fig. 2A). In the mouse model aimed at studying post-CIA hair regrowth, we synchronized all mice under the same damage-response pathway by delaying the start of vehicle, N-CM, and H-CM treatment until day 15 p.d.. This deliberation allowed for a more precise investigation into the influence of post-CIA DPSC-CM application on hair regrowth after CIA. Microstructure observation of the hair surface further revealed the differences between the primary and secondary recovery hair (Fig. 5A).

In this study, we investigated the effect of DPSC-CM on molecular damage-response pathways in CYP-treated mice (Fig. 2A). Notably, DPSC-CM suppressed apoptosis (Fig. 4A, B) and downregulated the Caspase-3/7 activities in the HFs and skin tissue, respectively, of CYP-treated mice (Fig. 4C). Moreover, H-CM negatively regulated the Caspase-3 mRNA expression (Fig. 4D). These data reveal the cytoprotective role of DPSC-CM in CIA by downregulating apoptosis through Caspase-3/7. p53 plays a central role in CIA, and ﻿a p53 null mutation in mice was associated with the prevention of CIA and apoptosis in the HF keratinocytes [33]. However, in the present study, mRNA expression of p53 did not decrease in the N-CM- and H-CM-treated groups (Fig. 4D), possibly because the day 16 p.d. detection of mRNA expression levels was not an appropriate time point. Additionally, this result reflects mRNA expression levels in the entire skin tissue and may not accurately reflect changes in the expression level in the HF. Therefore, a more appropriate and accurate sampling time point and method, respectively, will be necessary in future studies.

The hair-growth effects of N-CM can be enhanced by an increase of relevant growth factors such as VEGF, bFGF, and PDGF under hypoxic conditions in the dermal papilla and outer root sheath cells, organ culture, and mice [19, 20]. A previous study revealed that hypoxia enhanced the hair growth-promoting effects of embryonic stem cell-derived MSC-CM via NADPH oxidase 4 [19]. In another study, hypoxia treatment of adipose-MSCs promoted the growth of dermal papilla cells via hypoxia-inducible factor-1α and ERK1/2 signaling pathways [20]. In the present study, the proportion of post-CIA H-CM-treated mice with > 90% normal hair was nearly twice that of vehicle- or N-CM-treated mice between days 50 and 59 p.d. (Fig. 5D). Thus, even in CYP-treated mice, H-CM may still exert stronger hair growth effects than N-CM.

Here, DPSC-CM was found to suppress proliferation in certain carcinoma cell lines in vitro, and did not promote the squamous cell carcinoma (SCC-VII) tumor growth rate in C3H/HeNCrl mice (Fig. 7A–C). Furthermore, N-CM demonstrated a more potent anti-cancer effect than H-CM in HeLa and SAS cells (Fig. 7A). Our results indicate the potential for DPSC-CM to safely treat CIA, although the interaction between DPSC-CM and carcinoma cells in humans needs further exploration. A previous study revealed that DPSC-CM had significant apoptotic and growth inhibitory effects on colorectal cancer cells via the MAPKinase and apoptosis signaling pathways [35], whereas another study revealed that DPSC-CM did not affect the proliferation and chemosensitivity of human oral squamous cell carcinoma cell lines and tumor growth in nude mice [36]. Additionally, CM collected from other sources of MSC such as human adipose-derived MSCs reportedly had anti-tumorigenic effects on B16 melanoma cells in vitro and in vivo [37]. Conversely, another study revealed that DPSC-CM can increase the carcinogenic properties of oral and breast cancers and melanoma cells in vitro [38]. The effect of MSC-CM on cancer cells may vary depending on the carcinoma cell lines, preparation protocol of CM, and MSC donors. Therefore, investigating the effects of DPSC-CM on a wider range of carcinoma cell lines and its mechanisms is crucial.

The results should be evaluated considering the limitations of this study. The data were based on a CYP-induced alopecia model; thus, the effect of DPSC-CM on CIA may vary when chemotherapeutic agents are changed or combined. The study at the level of molecular damage-response pathways in CYP-treated mice used the entire skin tissue; therefore, the results of Caspase-3/7 activity and mRNA expression reflect those within the entire mouse skin tissue, not just the HFs.

## Conclusions

DPSC-CM demonstrated significant cytoprotective effects against CYP-mediated cytotoxicity in NHEKs and mice; moreover, post-CIA H-CM application may accelerate hair regrowth and improve hair quality. Furthermore, DPSC-CM suppressed proliferation in certain carcinoma cell lines in vitro and did not promote the SCC-VII tumor growth rate in mice. Therefore, this study encourages the further exploration of DPSC-CM and H-CM as a potential cytoprotective agent and hair regrowth stimulant, respectively, for CIA.

### Supplementary Information

**Additional file 1: Table S1.** Description of data: Primers for RT-qPCR.

**Additional file 2: Fig. S1.** Description of data: Quantitative analysis of scanning electron microscope images. (**A**) Diameter of primary and secondary recovery hair shafts. Data are presented as mean ± SEM. ****P < 0.0001. (**B**) Proportion of primary and secondary recovery hair shafts with surface cracks. (**A, B**) The dorsa of 18 mice from the three groups (vehicle, N-CM, and H-CM) were divided equally into six regions, and a hair shaft from each region was selected randomly as a representative sample for a total of 108 hair shafts. The sample size of the primary recovery hair was 24 and that of the secondary recovery hair was 84.

## Data Availability

The data that support the findings of this study are available within the article and the Supplementary Information. All other raw data are available upon reasonable request from the corresponding author.

## Abbreviations

CIA: Chemotherapy-induced alopecia
CYP: Cyclophosphamide
DPSC: Dental pulp stem cells
CM: Conditioned medium
LDH: Lactate dehydrogenase
RT-qPCR: Reverse transcription-quantitative polymerase chain reaction
SCC-VII: Squamous cell carcinoma
pCIA: Permanent CIA
MSCs: Mesenchymal stem cells
KGF: Keratinocyte growth factor
VEGF: Vascular endothelial growth factor
HGF: Hepatocyte growth factor
PDGF: Platelet-derived growth factor
IGF-I: Insulin-like growth factor I
bFGF: Basic fibroblast growth factor
HF: Hair follicle
N: Normoxic
H: Hypoxic
NHEKs: Normal human epidermal keratinocytes
DMEM: Dulbecco’s Modified Eagle’s Medium
PBS: Phosphate-buffered saline
KSFM: Keratinocyte serum-free medium
TUNEL: Terminal deoxynucleotidyl transferase dUTP nick end labeling
SEM: Scanning electron microscope
p.d.: Post-depilation

## References

[1] McGarvey EL, Baum LD, Pinkerton RC, Rogers LM (2001). Psychological sequelae and alopecia among women with cancer. Cancer Pract.

[2] Trüeb RM (2009). Chemotherapy-induced alopecia. Semin Cutan Med Surg.

[3] Chan J, Adderley H, Alameddine M, Armstrong A, Arundell D, Fox R (2021). Permanent hair loss associated with taxane chemotherapy use in breast cancer: a retrospective survey at two tertiary UK cancer centres. Eur J Cancer Care (Engl).

[4] Kang D, Kim IR, Choi EK, Im YH, Park YH, Ahn JS (2019). Permanent chemotherapy-induced alopecia in patients with breast cancer: a 3-year prospective cohort study. Oncologist.

[5] Lyakhovitsky A, Segal O, Maly A, Zlotogorski A, Barzilai A (2022). Permanent chemotherapy-induced alopecia after hematopoietic stem cell transplantation treated with low-dose oral minoxidil. JAAD Case Rep.

[6] Basilio FM, Brenner FM, Werner B, Rastelli GJ (2015). Clinical and histological study of permanent alopecia after bone marrow transplantation. An Bras Dermatol.

[7] Wikramanayake TC, Haberland NI, Akhundlu A, Laboy Nieves A, Miteva M (2023). Prevention and treatment of chemotherapy-induced alopecia: what is available and what is coming?. Curr Oncol.

[8] Wang S, Yang T, Shen A, Qiang W, Zhao Z, Zhang F (2021). The scalp cooling therapy for hair loss in breast cancer patients undergoing chemotherapy: a systematic review and meta-analysis. Support Care Cancer.

[9] Paus R, Haslam IS, Sharov AA, Botchkarev VA (2013). Pathobiology of chemotherapy-induced hair loss. Lancet Oncol.

[10] Gronthos S, Brahim J, Li W, Fisher LW, Cherman N, Boyde A (2002). Stem cell properties of human dental pulp stem cells. J Dent Res.

[11] Kichenbrand C, Velot E, Menu P, Moby V (2019). Dental pulp stem cell-derived conditioned medium: an attractive alternative for regenerative therapy. Tissue Eng Part B Rev.

[12] Vizoso FJ, Eiro N, Cid S, Schneider J, Perez-Fernandez R (2017). Mesenchymal stem cell secretome: toward cell-free therapeutic strategies in regenerative medicine. Int J Mol Sci.

[13] Pawitan JA (2014). Prospect of stem cell conditioned medium in regenerative medicine. Biomed Res Int.

[14] Gunawardena TNA, Masoudian Z, Rahman MT, Ramasamy TS, Ramanathan A, Abu Kasim NH (2019). Dental derived stem cell conditioned media for hair growth stimulation. PLoS ONE.

[15] Yamaguchi S, Shibata R, Yamamoto N, Nishikawa M, Hibi H, Tanigawa T (2015). Dental pulp-derived stem cell conditioned medium reduces cardiac injury following ischemia-reperfusion. Sci Rep.

[16] Fujio M, Xing Z, Sharabi N, Xue Y, Yamamoto A, Hibi H (2017). Conditioned media from hypoxic-cultured human dental pulp cells promotes bone healing during distraction osteogenesis. J Tissue Eng Regen Med.

[17] Hirata M, Ishigami M, Matsushita Y, Ito T, Hattori H, Hibi H (2016). Multifaceted therapeutic benefits of factors derived from dental pulp stem cells for mouse liver fibrosis. Stem Cells Transl Med.

[18] Izumoto-Akita T, Tsunekawa S, Yamamoto A, Uenishi E, Ishikawa K, Ogata H (2015). Secreted factors from dental pulp stem cells improve glucose intolerance in streptozotocin-induced diabetic mice by increasing pancreatic β-cell function. BMJ Open Diabetes Res Care.

[19] Jeon SH, Kim H, Sung JH (2023). Hypoxia enhances the hair growth-promoting effects of embryonic stem cell-derived mesenchymal stem cells via NADPH oxidase 4. Biomed Pharmacother.

[20] Wang Q, Zhou M, Zhang H, Hou Z, Liu D (2023). Hypoxia treatment of adipose mesenchymal stem cells promotes the growth of dermal papilla cells via HIF-1α and ERK1/2 signaling pathways. Int J Mol Sci.

[21] Narita K, Fukuoka H, Sekiyama T, Suga H, Harii K (2020). Sequential scalp assessment in hair regeneration therapy using an adipose-derived stem cell-conditioned medium. Dermatol Surg.

[22] Shin H, Ryu HH, Kwon O, Park BS, Jo SJ (2015). Clinical use of conditioned media of adipose tissue-derived stem cells in female pattern hair loss: a retrospective case series study. Int J Dermatol.

[23] Shimizu Y, Ntege EH, Sunami H, Inoue Y (2022). Regenerative medicine strategies for hair growth and regeneration: a narrative review of literature. Regen Ther.

[24] Ramdasi S, Tiwari SK (2016). Human mesenchymal stem cell-derived conditioned media for hair regeneration applications. J Stem Cells.

[25] Chouaib B, Cuisinier F, Collart-Dutilleul PY (2022). Dental stem cell-conditioned medium for tissue regeneration: optimization of production and storage. World J Stem Cells.

[26] Matsumura-Kawashima M, Ogata K, Moriyama M, Murakami Y, Kawado T, Nakamura S (2021). Secreted factors from dental pulp stem cells improve Sjögren's syndrome via regulatory T cell-mediated immunosuppression. Stem Cell Res Ther.

[27] Paus R, Handjiski B, Eichmüller S, Czarnetzki BM (1994). Chemotherapy-induced alopecia in mice. Induction by cyclophosphamide, inhibition by cyclosporine A, and modulation by dexamethasone. Am J Pathol.

[28] Hendrix S, Handjiski B, Peters EM, Paus R (2005). A guide to assessing damage response pathways of the hair follicle: lessons from cyclophosphamide-induced alopecia in mice. J Investig Dermatol.

[29] Ohnemus U, Unalan M, Handjiski B, Paus R (2004). Topical estrogen accelerates hair regrowth in mice after chemotherapy-induced alopecia by favoring the dystrophic catagen response pathway to damage. J Investig Dermatol.

[30] Haslam IS, Zhou G, Xie G, Teng X, Ao X, Yan Z (2021). Inhibition of Shh signaling through MAPK activation controls chemotherapy-induced alopecia. J Investig Dermatol.

[31] Müller-Röver S, Handjiski B, van der Veen C, Eichmüller S, Foitzik K, McKay IA (2001). A comprehensive guide for the accurate classification of murine hair follicles in distinct hair cycle stages. J Investig Dermatol.

[32] Bodó E, Tobin DJ, Kamenisch Y, Bíró T, Berneburg M, Funk W (2007). Dissecting the impact of chemotherapy on the human hair follicle: a pragmatic in vitro assay for studying the pathogenesis and potential management of hair follicle dystrophy. Am J Pathol.

[33] Botchkarev VA, Komarova EA, Siebenhaar F, Botchkareva NV, Komarov PG, Maurer M (2000). p53 is essential for chemotherapy-induced hair loss. Cancer Res.

[34] Fan TJ, Han LH, Cong RS, Liang J (2005). Caspase family proteases and apoptosis. Acta Biochim Biophys Sin (Shanghai).

[35] Nikkhah E, Kalalinia F, Asgharian Rezaee M, Tayarani-Najaran Z (2021). Suppressive effects of dental pulp stem cells and its conditioned medium on development and migration of colorectal cancer cells through MAPKinase pathways. Iran J Basic Med Sci.

[36] Hanyu S, Sakuma K, Tanaka A (2019). A study on the effect of human dental pulp stem cell conditioned medium on human oral squamous cell carcinoma cell lines. J Hard Tissue Biol.

[37] Lee JH, Park CH, Chun KH, Hong SS (2015). Effect of adipose-derived stem cell-conditioned medium on the proliferation and migration of B16 melanoma cells. Oncol Lett.

[38] Raj AT, Kheur S, Bhonde R, Mani VR, Baeshen HA, Patil S (2021). Assessing the effect of human dental pulp mesenchymal stem cell secretome on human oral, breast, and melanoma cancer cell lines. Saudi J Biol Sci.

