# Supplementary material for: Cytoprotective role of human dental pulp stem cell-conditioned medium in chemotherapy-induced alopecia
Source: Stem Cell Res Ther. 2024 Mar 18;15:84. doi: 10.1186/s13287-024-03695-3 (PMC10949570; doi:10.1186/s13287-024-03695-3)
Supplement: Supplementary file 1 — Additional file 1: Table S1. Description of data: Primers for RT-qPCR. [file 13287_2024_3695_MOESM1_ESM.docx]

**Additional file 1: Table S1. Primers for RT-qPCR**

| **Genes (Mus musculus)** | **Primer sequence (5′–3′)** | |
| --- | --- | --- |
| *Gapdh* | forward | AATTCAACGGCACAGTCAAGG |
|  | reverse | TGTTAGTGGGGTCTCGCTCC |
| *p53* | forward | CGCCGACCTATCCTTACCAT |
|  | reverse | CACAAACACGAACCTCAAAGC |
| *Casp3* | forward | TCTGACTGGAAAGCCGAAAC |
|  | reverse | TGGCAAGCCATCTCCTCAT |

Abbreviations: *Gapdh*, glyceraldehyde-3-phosphate dehydrogenase; *p53*, transformation related protein 53; *Casp3*, caspase 3.
