# Supplementary material for: Cytoprotective role of human dental pulp stem cell-conditioned medium in chemotherapy-induced alopecia
Source: Stem Cell Res Ther. 2024 Mar 18;15:84. doi: 10.1186/s13287-024-03695-3 (PMC10949570; doi:10.1186/s13287-024-03695-3)
Supplement: Supplementary file 2 — Additional file 2: Fig. S1. Description of data: Quantitative analysis of scanning electron microscope images. (A) Diameter of primary and secondary recovery hair shafts. Data are presented as mean ± SEM. ****P < 0.0001. (B) Proportion of primary and secondary recovery hair shafts with surface cracks. (A, B) The dorsa of 18 mice from the three groups (vehicle, N-CM, and H-CM) were divided equally into six regions, and a hair shaft from each region was selected randomly as a representative sample for a total of 108 hair shafts. The sample size of the primary recovery hair was 24 and that of the secondary recovery hair was 84. [file 13287_2024_3695_MOESM2_ESM.docx]

**
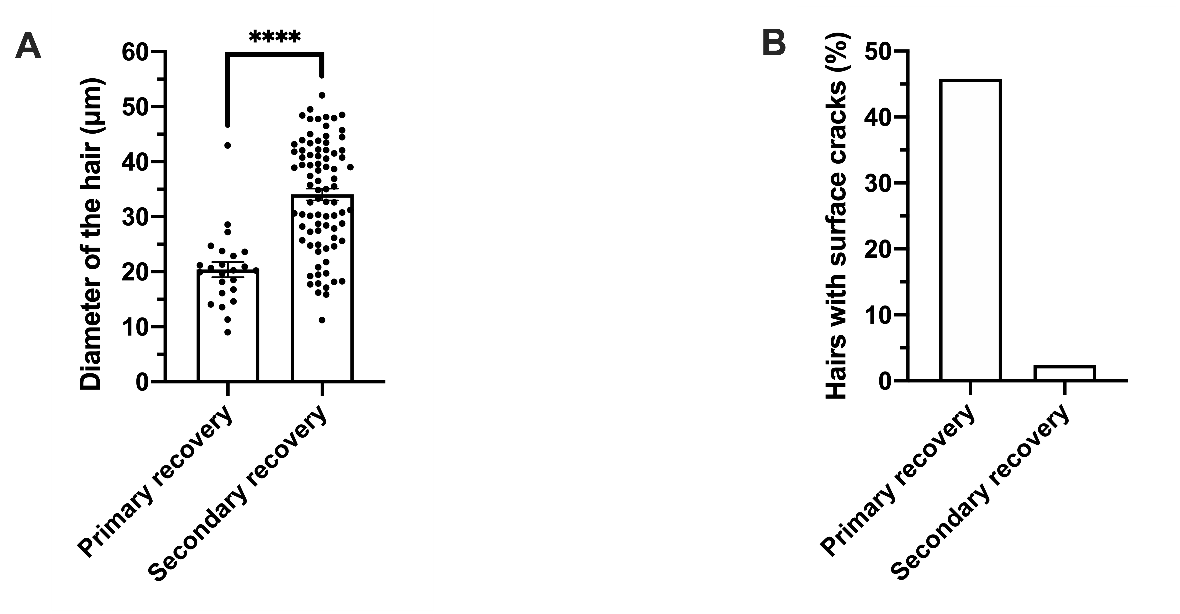
**

**Additional file 2: Fig. S1. Quantitative analysis of scanning electron microscope images**

(**A**) Diameter of primary and secondary recovery hair shafts. Data are presented as mean ± SEM. ^****^*P*<0.0001. (**B**) Proportion of primary and secondary recovery hair shafts with surface cracks. (**A, B**) The dorsa of 18 mice from the three groups (vehicle, N-CM, and H-CM) were divided equally into six regions, and a hair shaft from each region was selected randomly as a representative sample, for a total of 108 hair shafts. The sample size of the primary recovery hair was 24, and that of the secondary recovery hair was 84.
